# Modeling Spatial Distribution of *Sergentomyia minuta* (Diptera: Psychodidae) and Its Potential Implication in Leishmaniasis Transmission in Morocco

**DOI:** 10.18502/jad.v14i1.2700

**Published:** 2020-03-31

**Authors:** Morocco Mohamed Daoudi, Samia Boussaa, Ali Boumezzough

**Affiliations:** 1 Laboratory of Ecology and Environment (L2E), (URAC 32), Cadi Ayyad University, Faculty of Sciences Semlalia, Marrakesh, Morocco; 2 ISPITS-Higher Institute of Nursing and Technical Health Occupations, Ministry of Health, Marrakesh, Morocco

**Keywords:** *Sergentomyia minuta*, Ecological niche modeling, MaxEnt, Morocco

## Abstract

**Background:**

Leishmaniases are parasitic diseases caused by *Leishmania* species and transmitted by the bite of sand flies. The genus *Lutzomyia* and *Phlebotomus* of sand flies are known to be the responsible vector for transmitting almost all *Leishmania* species to humans. The detection of *Leishmania* DNA in species of the genus *Sergentomyia*, in different regions, suggests their likely role in *Leishmania* transmission.

**Methods:**

Our objective was to determine the potential geographical distribution of *Sergentomyia minuta,* the most dominant *Sergentomyia* species in Morocco, using ecological niche modeling.

**Results:**

The results showed the widespread geographical distribution of *S. minuta* in Morocco, specifically in northern and central Morocco where visceral and cutaneous leishmaniasis foci occur. There were six abiotic factors affecting the distribution of *S. minuta* whose annual precipitation, precipitation seasonality and precipitation of driest month as the most important ecological variables of the model.

**Conclusion:**

A positive statistical correlation between human leishmaniasis cases and *S. minuta* abundance was noted suggesting the potential involvement of *S. minuta* in local *Leishmania* transmission cycles.

## Introduction

Phlebotomine sand flies (Diptera: Psychodidae) are small nocturnal insects of which only the female is a hematophagous. Currently, about 1000 species of sand flies are distributed in almost all biogeographic regions of the world (1). These insects play a crucial role in the epidemiology of relevant diseases, some being of great veterinary and medical importance. Indeed, certain species of sand flies are vectors of various infectious and parasitic agents such as agents of canine and human leishmaniasis, bartonellosis and several arboviruses. Allergic reactions can also be caused by the exposure of sand fly bites, though is not linked to the spread of disease (2).

Leishmaniasis remains the most related diseases to sand flies. The genera *Phlebotomus*, *Lutzomyia* and *Sergentomyia* contain all hematophagous species of sand flies (3), with only the genus *Lutzomyia* in the New World, and *Phlebotomus* in the Old World responsible for transmitting almost all *Leishmania* species to humans (4, 5).

Like most countries around the Mediterranean, leishmaniases in Morocco are a serious public health problem. These affections are widely represented, from the Moroccan Rif Mountains to the palm groves of the Moroccan Anti-Atlas (6). Three epidemiological entities are known in Morocco: zoonotic cutaneous leishmaniasis due to *Leishmania major*, anthroponotic cutaneous leishmaniasis due to *L. tropica* and visceral and cutaneous leishmaniasis due to *L. infantum* (6).

Regarding disease transmission, only vectorial role of species of the genus *Phlebotomus* (namely, *Phlebotomus papatasi*, *P. sergenti*, *P. ariasi*, *P. longicuspis* and *P. perniciosus*) was investigated in Morocco (7–9); where sand fly fauna has 22 species including, nine species of the genus *Sergentomyia* (10): *Sergentomyia christophersi*, *S. clydei*, *S. africana*, *S. minuta*, *S. dreyfussi*, *S. schwetzi*, *S. antennata*, *S. fallax* and *S. lewisi*.

Considering the epidemiological status of leishmaniasis in Morocco, it is critically important to highlight the probable role as a vector for other sand fly species. Vector incrimination depends on the accumulation of evidence based mainly on its geographical and temporal abundance. According to many authors, *Sergentomyia* species are spread throughout Morocco, especially *S. minuta* and *S. fallax* (11, 12). *Sergentomyia minuta* is collected in urban as well as in rural area (13) up to 1,200m (14). It was considered as ubiquitous species in Morocco (15) with high density and a long activity period (16, 17).

In addition, *Leishmania* DNA was detected in species of the genus *Sergentomyia* in different regions suggesting their likely role in *Leishmania* transmission (18). Studies conducted in leishmaniasis foci in Iran, Mali and Portugal have reported the detection of *L. major* DNA in *Sergentomyia sintoni* (19), *S. darlingi* (20) and *S. minuta* (21). Earlier, it has also been isolated from *S. garnhami* in Kenya (22). Other reports also detected *L*. *donovani* DNA in *S. babu* in India (23) and more recently, *L. siamensis* detected in *S. gemmea* in Thailand (24). Moreover, several viruses have been isolated from sand flies of the genus *Sergentomyia* such as the *Saboya* virus, viruses RNA of ArD 95737 and ArD 111740 (25, 26), which has made it possible to suspect the vector role of *Sergentomyia* species (27).

Despite the presence and the abundance of *Sergentomyia* species in Morocco, no study has invested its potential involvement in leishmaniasis transmission cycles. Therefore, we used ecological niche modeling to identify the distribution of the most dominant species of *Sergentomyia* genus in Morocco and consequently discuss its potential involvement in local *Leishmania* transmission cycles.

## Materials and Methods

### Study area

**Fig. 1.**
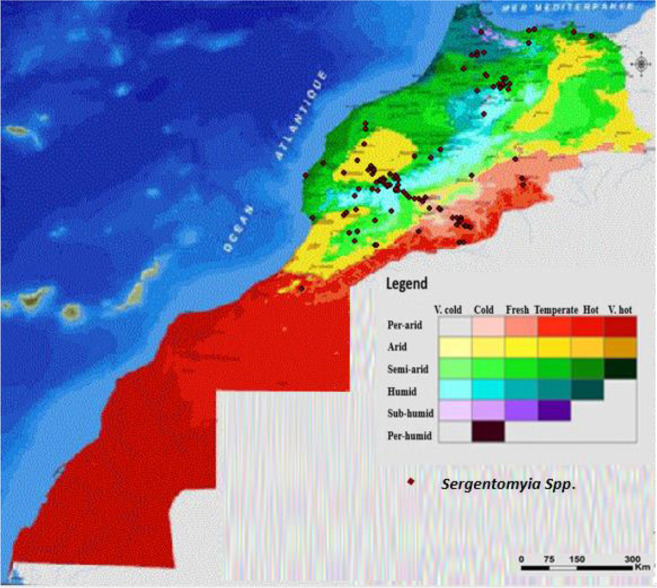
Distribution of the *Sergentomyia* spp in Morocco according to bioclimate map

Morocco is a northern African country, bordering the North Atlantic Ocean and the Mediterranean Sea. The current study covered sand fly sampling in 190 localities with altitude ranges between sea level and up to 2123m above sea (Fig. 1). These localities covered mainly northern and central Morocco, where human cutaneous and visceral leishmaniases are endemic (28).

The climate in Morocco is mostly Mediterranean; however, seven bioclimatic regimes are occurred due to topographic differences across the country (Fig. 1). In 2014, the total human population in Morocco is about 33.7 million, with an urbanization rate of 60% (29).

### Occurrence data and environmental variables

Occurrence records of sand flies were collected from our field survey (published and unpublished data) and literature using the keyword “species name, Morocco” as a search word to retrieve all data archived in PubMed database. Thus, about 70% of the 190 localities were investigated by the Ecology and Environment Laboratory (L2E) team between 2005 and 2016. The rest of the localities were published observations of the investigations in Morocco (20 publications on PUBMED). The coordinates of all localities were registered with a global positioning system (GPS).

For ecological niche modeling, 19 climatic variables (Bio1-Bio19) were used with elevation precision (ALT) for each locality (Table 1). Altitude and bioclimatic data were obtained from the WorldClim project (http://www.worldclim.org), with a spatial resolution of 30 arc seconds (about 1km^2^).

**Table 1.** Description and sources of environmental variables collected for the model

**Environmental variables**	**Abbreviation**	**Unit**	**Source**
**Annual mean temperature**	Bio 1	°C	WorldClim
**Mean diurnal range (mean of monthly (max temperature–min temperature))**	Bio 2	°C	WorldClim
**Isothermality (BIO2/BIO7) (×100)**	Bio 3	_	WorldClim
**Temperature seasonality (standard deviation ×100)**	Bio 4	°C	WorldClim
**Max temperature of warmest month**	Bio 5	°C	WorldClim
**Min temperature of coldest month**	Bio 6	°C	WorldClim
**Temperature annual range (BIO5–BIO6)**	Bio 7	°C	WorldClim
**Mean temperature of wettest quarter**	Bio 8	°C	WorldClim
**Mean temperature of driest quarter**	Bio 9	°C	WorldClim
**Mean temperature of warmest quarter**	Bio 10	°C	WorldClim
**Mean temperature of coldest quarter**	Bio 11	°C	WorldClim
**Annual precipitation**	Bio 12	mm	WorldClim
**Precipitation of wettest month**	Bio 13	mm	WorldClim
**Precipitation of driest month**	Bio 14	mm	WorldClim
**Precipitation seasonality (coefficient of variation)**	Bio 15	mm	WorldClim
**Precipitation of wettest quarter**	Bio 16	mm	WorldClim
**Precipitation of driest quarter**	Bio 17	mm	WorldClim
**Precipitation of warmest quarter**	Bio 18	mm	WorldClim
**Precipitation of coldest quarter**	Bio 19	mm	WorldClim
**Elevation**	ALT	m	GTOPO30

### Construction of the model

The modeling was performed using Maximum entropy (MaxEnt) version 3.3.3 (30), which uses an optimization procedure comparing the presence of the species with the characteristics of the environment, based on the principle of maximum entropy. This program allows for inference from incomplete information despite limited occurrence data (31–33).

Distribution data for the sand fly species and environmental data were imported into MaxEnt v. 3. 3. 3 (31), a total of 15 model replicates were run, with 30% of the points of presence used to test the model and 70% for model construction (34).

### Model Evaluation

To evaluate the quality of model produced by MaxEnt, we analyzed the Operating Characteristics Curve Receiver Operating Characteristic (ROC) which assigns a unique value according to the model performance Area Under the Curve (AUC).

**Fig. 2.**
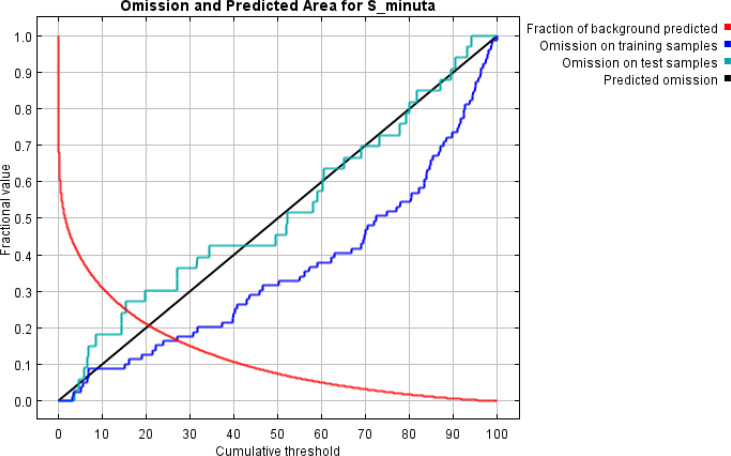
Analysis of the omission / commission of *Sergentomyia minuta*

**Fig. 3.**
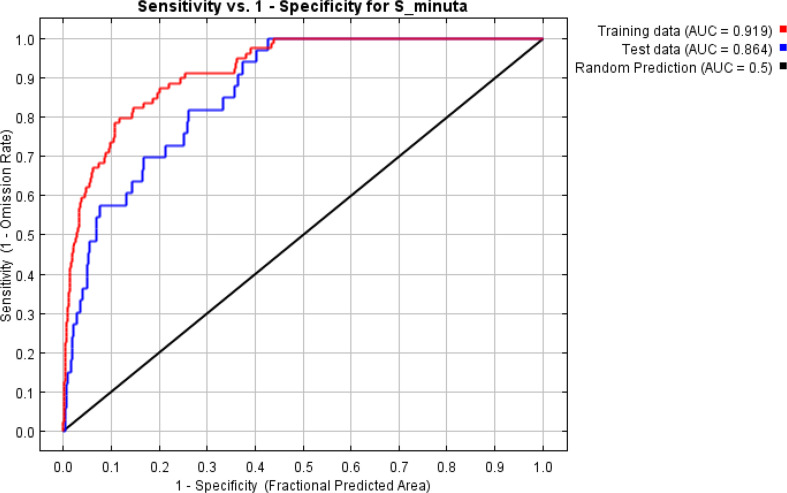
Sensitivity and performance of the model for *Sergentomyia minuta* species

ROC analysis is a measure of sensitivity, which corresponds to the true positive rate (no error of omission), compared to the false positive rate (superfluous forecast error). Also, the ROC analysis evaluates the ability of the model to correctly predict the occurrence of the species. The closer the AUC value is to 1, the closer the correlation of the model (31) (Figs. 2, 3). The importance of the variables in explaining the potential geographic distribution of *S. minuta* was estimated using the Jackknife Environmental Variables Importance Test (31) (Table 2), which assesses the relative contribution (%) of the variables used to generate the distribution model produced by MaxEnt.

**Table 2.** Analysis of the contribution of the most important ecological variables of the model

**Variable**	**Percent contribution (%)**	**AUC without the variable**	**AUC with only the variable**
**Bio 12**	41.2	0.917	0.867
**Bio 15**	23.9	0.922	0.892
**Bio 14**	9.4	0.916	0.859
**Bio 4**	5.9	0.914	0.890
**Bio 19**	5.5	0.914	0.906
**Bio 3**	4.8	0.914	0.868

### Statistical analysis

The Chi-squared test (*χ*^2^) was used to analyze the correlation between variation of *S. minuta* abundance and the presence/absence of human leishmaniasis cases (cutaneous leishmaniasis by *L. tropica* and visceral leishmaniasis by *L. infantum*) in all sampling sites.

Epidemiological data (human cutaneous and visceral leishmaniasis cases) were obtained from official Moroccan Ministry of Health reports (28).

## Results

In this study, we assembled 190 sampling sites for sand fly species. Five *Sergentomyia* species were noted: *Sergentomyia minuta* (N= 150), *S. fallax* (N= 110), *S. dreyfussi* (N= 57), *S. africana* (N= 10) and *S. christophersi* (N= 15). *Phlebotomus* species were also recorded, specifically *Phlebotomus sergenti* (N= 102), *P. papatasi* (N= 82), *P. longicuspis* (N= 78), *P. perniciosus* (N= 76) and *P. ariasi* (N= 43), while *P. alexandri* (N= 09), *P. kazeruni* (N= 03), *P. langeroni* (N= 02), and *P. mariae* (N= 02) were noted with very low reporting.

*Sergentomyia minuta* was the most predominant species and it was collected in the 150/190 sampling sites between 14 and 2123m (Table 3). To identify habitat suitability and potential limiting factor of *S. minuta*, all ecological variables were analyzed. There were 6/19 ecological variables (Bio 3, Bio 4, Bio 12, Bio 14, Bio 15, Bio 19), which were the most important factors affecting the distribution of *S. minuta* in the study area.

**Table 3.** Description of *Sergentomyia* species on sampling sites

***Sergentomyia spp.***	**Total of specimens**	**Number of stations occupied**	**Altitude range (m)**
***S. minuta***	6746	150	[14–2123]
***S. fallax***	5582	110	[14–1351]
***S. dreyfussi***	474	57	[282–1309]
***S. africana***	43	10	[402–1340]
***S. christophersi***	99	15	[282–1500]

**Fig. 4.**
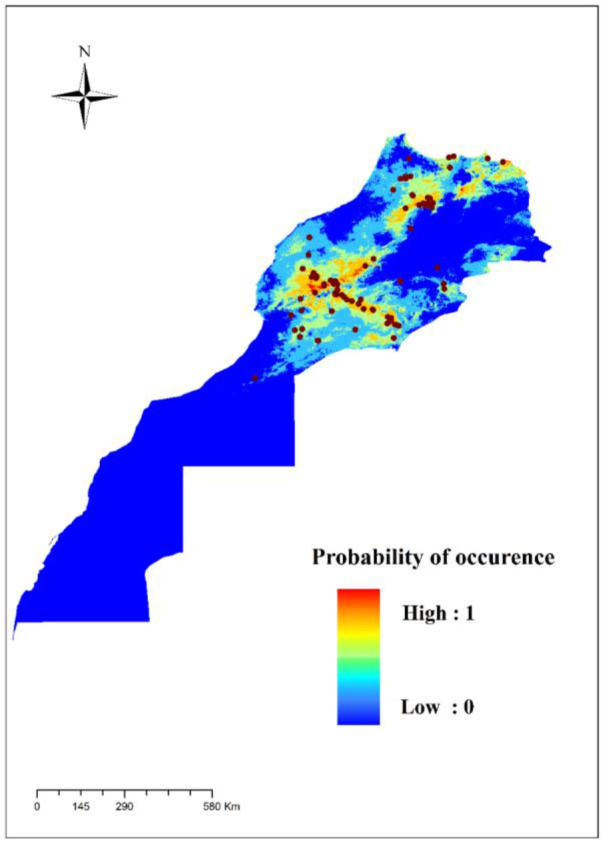
Estimation of the distribution of *Sergentomyia minuta* species in Morocco by MaxEnt

Figure 4 shows a representation of the MaxEnt model for the *S. minuta*. Warmer colors show areas with better predicted conditions. The white dots indicate the presence locations used for training, while the purple dots show the locations of the tests.

Since 1995, leishmaniases are notifiable diseases in Morocco (Ministerial decree n°683-95). All human cases are listed in leishmaniasis epidemiological information collection system and published yearly. We used data recorded of CL and VL between 2005 and 2016 (28) corresponding to 190 localities.

Statistical analysis of the presence and absence of leishmaniasis cases (cutaneous CL and visceral leishmaniasis VL) and the distribution of *S. minuta* in Morocco showed a positive correlation between VL by *L. infantum* and *S. minuta* abundance (r= 0.211) and between CL by *L. tropica* and *S. minuta* abundance (r= 0.166).

According to Chi-squared test (*χ*^2^) test, *S. minuta* was more associated to VL distribution (*χ*^2^ = 7,166, ddl= 2, P= 0,028) compared to CL (*χ*^2^ = 3,256, ddl= 2, P= 0,196).

## Discussion

Morocco is located in the subtropical zone. It benefits from a Mediterranean climate, characterized by a dry warm season, nuanced by three essential influences: the progressive remoteness of the Atlantic coast, the altitude and the approach of the southern desert (36). The geographical location of Morocco is a major factor of its bioclimatic diversity which favors the abundance and the diversity of phlebotomine species; including *Sergentomyia* species that can tolerate the aridity (37–39). Thus, a considerable specific richness in Morocco for *Sergentomyia* genus counter to the others Mediterranean’s regions such as Tunisia, where only six species of *Sergentomyia* have been reported (40–42).

*Sergentomyia* species were collected in different environments but are rather subservient to the extradomestic environment (43). This is related to their trophic preferences, and their adaptability to dry and open habitats. Thus, they find suitable conditions in wild environments.

According to WHO (43), sand flies of the genus *Sergentomyia* have long been known to feed on reptiles. They are involved in the transmission of *Sauroleishmania*, of reptiles in the Old World. They do not present an epidemiological risk for humans (44), although some species can feed on humans (45). Recent work on *Sergentomyia* spp shows that these species can be infected by human *Leishmania* spp. and the blood meal analysis shows that it is a mammalian blood, including humans (18). *Leishmania tropica* DNA was detected from *S. ingrami* and *S. hamoni* collected in Ghana (46). All of these results raise questions about the role of *Sergentomyia* spp. as a possible vector of *Leishmania* spp. which has a great impact on human and animal health.

Among all *Sergentomyia* species, *S. minuta* is widely distributed around the Mediterranean area. It is the most abundant *Sergentomyia* species in the present study (Table 3) and early in Morocco (12).

In Greece, the *Sergentomyia* genus represented 84% of the total sand flies fauna with 38% of *S. minuta* (47). In France, Rioux et al. (48) reported a percentage of 73.7% for both genus and species. In Tunisia, it was noted that *Sergentomyia* accounted for 70% of sand flies, of which 41.5% belonged to the species *S. minuta* (49). In Algeria, Belazzoug et al. (50) found this species with a rate of 39.75% of total specimens.

Regarding its potential vector role, Periera et al. (51) have isolated *L. infantum* from *S. minuta*, and blood meal analysis has shown that it is human blood. In Italy, recently, Latrofa et al. (52) detected *L. infantum* DNA in *S. minuta* from endemic area of canine leishmaniasis. *S. minuta* was also naturally infected with *L. major* in Portugal (21) and in Tunisia (53). In addition, Maia et al. (54) detected *Leishmania* spp. in *S. minuta* collected in southern Portugal; while Bravo-Barriga et al. (55) identified *Leishmania* DNA in *S. minuta* of Spain.

In Morocco, though the well-known role of *Phlebotomus papatasi*, *P. sergenti* and *Larroussius* species (*P. perniciosus*, *P. longicuspis* and *P. ariasi*), as competent vector of *L. major*, *L. tropica* and *L. infantum*, respectively (56), that of *S. minuta* needs further investigation.

Ecological niche modelling has been widely used in order to identify the probability of sand fly species occurrence (57). To the best of our knowledge, the present study constitutes a first modeling of *Sergentomyia* species. AUC values of our model were greater than 0.9 (Table 2) indicating that the model performed has a good robustness (31).

In the 190 localities used for the model, we noted the coexistence of *Sergentomyia* species and *Phlebotomus* species including the proven vectors of leishmaniasis forms in Morocco: *P. papatasi*, *P. sergenti*, *P. perniciosus*, *P. ariasi* and *P. longicuspis*. According to the region, *P. sergenti* and *P. papatasi* were more abundant in the central Morocco, while *P. perniciosus* was more recorded in northern Morocco.

The presence and the abundance of *S. minuta* in the endemic area of leishmaniasis in Morocco can be the most important criteria for its possible vectorial incrimination. Our results showed the widespread geographical distribution of *S. minuta* in Morocco, specifically in northern and central Morocco (Fig. 4) where VL and CL foci occur (16, 58).

In addition, a positive correlation between visceral leishmaniasis (r= 0.211) and cutaneous leishmaniasis (r= 0.166) with *S. minuta* abundance was noted and confirm this *S. minuta*-*Leishmania* spatial overlapping.

Sand fly behaviors can also determine its vector implication. *S. minuta* presented an anthropophilic behavior in Portugal (54). In Morocco, S. *minuta* is a ubiquitous species (13) which shows an adaptation and an important ecological tolerance. It was collected in the different bioclimatic floors with preference to a semiarid climate and altitudes between 800 to 1,000m (12). In addition, the seasonal distribution of *S. minuta* showed abundance from May to September in sub-humid and semi-arid stages in Morocco (15). In arid bioclimate, it was active during two periods, October–November and April–May–June (59). This long period of *S. minuta* activity favors the *S. minuta*-*Leishmania* temporal overlapping in these areas.

Sand flies of Morocco, including *Sergentomyia* species, were found highly dependent on environmental conditions; and their abundance and geographical distribution were affected by several ecological variables (59–62). According to our model, annual precipitation (Bio 12) and precipitation seasonality (Bio 15) were significantly more important in the distribution of *S. minuta* in Morocco (Table 2). Precipitation plays an important role in sand flies’ life cycle (63). Modeling approach of Chargaff et al. (57), found that annual precipitation has a significant role in distribution of *Leishmania* vector species in Mediterranean basin (57).

*S. minuta* is recorded in arid region of Tunisia with an annual precipitation between 88 and 157mm (53), also in Portugal with an annual precipitation equal to 730mm (64). In the present study, it prefers regions with an annual precipitation (Bio 12) between: 50–1100mm, and precipitation seasonality (Bio 15) between: 30–100mm (Supporting Information S1).

## Conclusion

Effective vector control requires accurate vector identification. The spatial and temporal overlapping of *S. minuta* with *Leishmania* species seemed to present the important criteria necessary to be incriminated as a potential vector of the *Leishmania* species in Morocco. Hence, the present study opened a debate on the potential role of *Sergentomyia* species, especially *S. minuta* in the transmission of leishmaniasis in Morocco. Hypothesis deserves further researches including, but not limited to, protozoan isolation from engorged specimens as well as experimental transmission.
